# The initial age‐associated decline in early T‐cell progenitors reflects fewer pre‐thymic progenitors and altered signals in the bone marrow and thymus microenvironments

**DOI:** 10.1111/acel.13870

**Published:** 2023-05-23

**Authors:** Jayashree Srinivasan, Anusha Vasudev, Carolyn Shasha, Hilary J. Selden, Encarnacion Perez, Bonnie LaFleur, Shripad A. Sinari, Andreas Krueger, Ellen R. Richie, Lauren I. R. Ehrlich

**Affiliations:** ^1^ Department of Molecular Biosciences The University of Texas at Austin Austin Texas United States; ^2^ Department of Epigenetics and Molecular Carcinogenesis The University of Texas MD Anderson Cancer Center Houston Texas United States; ^3^ Vaccine and Infectious Disease Division Fred Hutchinson Cancer Center Seattle Washington United States; ^4^ Center for Biomedical Informatics and Statistics The University of Arizona Tucson Arizona United States; ^5^ Molecular Immunology Justus‐Liebig‐University Giessen Giessen Germany; ^6^ Department of Oncology Livestrong Cancer Institutes, Dell Medical School at The University of Texas at Austin Austin Texas United States

**Keywords:** aging, early T‐cell progenitors, hematopoiesis, thymus involution

## Abstract

Age‐related thymus involution results in decreased T‐cell production, contributing to increased susceptibility to pathogens and reduced vaccine responsiveness. Elucidating mechanisms underlying thymus involution will inform strategies to restore thymopoiesis with age. The thymus is colonized by circulating bone marrow (BM)‐derived thymus seeding progenitors (TSPs) that differentiate into early T‐cell progenitors (ETPs). We find that ETP cellularity declines as early as 3 months (3MO) of age in mice. This initial ETP reduction could reflect changes in thymic stromal niches and/or pre‐thymic progenitors. Using a multicongenic progenitor transfer approach, we demonstrate that the number of functional TSP/ETP niches does not diminish with age. Instead, the number of pre‐thymic lymphoid progenitors in the BM and blood is substantially reduced by 3MO, although their intrinsic ability to seed and differentiate in the thymus is maintained. Additionally, Notch signaling in BM lymphoid progenitors and in ETPs diminishes by 3MO, suggesting reduced niche quality in the BM and thymus contribute to the early decline in ETPs. Together, these findings indicate that diminished BM lymphopoiesis and thymic stromal support contribute to an initial reduction in ETPs in young adulthood, setting the stage for progressive age‐associated thymus involution.

## Graphical abstract

Early T‐cell progenitor (ETP) cellularity declines as early as 3 months (3MO) of age in the murine thymus, despite maintenance of functional thymus seeding progenitor niches. The early decline in ETPs reflects a sharp drop in circulating thymus seeding progenitors and upstream bone marrow lymphoid progenitors. Notch signaling declines in bone marrow lymphoid progenitors and in thymic ETPs by 3MO of age.

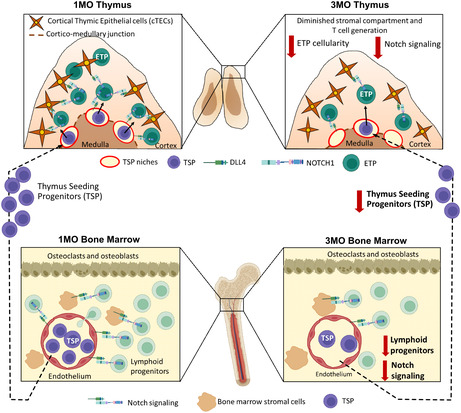

**Abbreviations**
BM: bone marrow
CLP: common lymphoid progenitor
CMP: common myeloid progenitor
cTECs: cortical epithelial cells
DLL4: Delta‐like Notch ligand 4
EC: endothelial cell
ETP: early T‐cell progenitor
FWB: FACS wash buffer
MO: months
MPP: multi‐potent progenitor
RBC: red blood cell
TSP: thymus seeding progenitor

## 1 INTRODUCTION

T cells develop in the thymus which provides a unique and complex stromal microenvironment essential for T‐cell lineage commitment, differentiation, and selection (Han & Zúñiga‐Pflücker, 2021; Yui & Rothenberg, 2014). Since thymocytes do not have the ability to self‐renew, the thymus periodically recruits bone marrow (BM)‐derived thymus seeding progenitors (TSPs) which are essential to maintain T‐cell production throughout life (Krueger et al., 2017). TSP entry is a well‐orchestrated multistep process mediated by the adhesion molecules P‐selectin, ICAM‐1, and VCAM‐1 and the chemokines CCL25 and CCL21, and supported by other niche factors, like KITL, supplied by thymic endothelial cells (Buono et al., 2016; Krueger et al., 2010; Rossi, Bryder, et al., 2005; Scimone et al., 2006; Zlotoff et al., 2010). After entering the thymus via vasculature at the cortico‐medullary junction, TSPs differentiate into highly proliferative CD4^−^CD8^−^CD44^hi^c‐kit^hi^ early T‐cell progenitors (ETPs) in the thymic cortex. Commitment of developing thymocytes to the T‐cell lineage depends on inductive signals provided by cortical thymic epithelial cells (cTECs) (Han & Zúñiga‐Pflücker, 2021; Petrie & Zúñiga‐Pflücker, 2007; Thompson & Zúñiga‐Pflücker, 2011; Yui & Rothenberg, 2014). In particular, activation of Notch signaling in ETPs via Delta‐like Notch ligand 4 (DLL4) expressed by cortical thymic epithelial cells (cTECs) is indispensable for T‐cell lineage commitment and differentiation (Hozumi, Mailhos, et al., 2008; Hozumi, Negishi, et al., 2008; Koch et al., 2008). Since ETPs give rise to all downstream T‐cell subsets, thymopoiesis and T‐cell development depend on both continued influx of TSPs into the thymus and the availability of functional stromal niches that support TSP entry and downstream ETP differentiation.

During age‐associated thymus involution, thymus size and cellularity undergo a progressive decline accompanied by reduced output of naïve T cells. Thymus involution is initiated early in life, by ~7 weeks of age in mice (Chen et al., 2009; Hale et al., 2006). Thus, we first asked if ETP cellularity is adversely affected during the earliest stages of thymus involution. Here, we find that the decline in ETP cellularity is initiated early, between 1MO and 3MO of age in mice, and progresses with advancing age. Thymus involution involves not only a reduction in thymocyte cellularity, but also a decline in the number and proliferative capacity of TECs, as well as changes in the composition and organization of the stromal compartment (Baran‐Gale et al., 2020; Chinn et al., 2012; Gray et al., 2007; Griffith et al., 2012; Lepletier et al., 2019). TEC degeneration is a key driver of age‐associated thymic involution, as sustaining or restoring TECs prevents or reverses involution, respectively (Bredenkamp et al., 2014; Chen et al., 2009; Garfin et al., 2013; Klug et al., 2000). Given that thymic stromal niche factors play a key role in recruitment of TSPs, and thymic stromal signals are required for ETP survival, proliferation, and T‐lineage commitment, we anticipated the early age‐associated decline in ETPs would be driven by changes in the thymic microenvironment.

Here, we used several approaches to determine whether the early loss of ETPs is due to age‐related changes in availability of functional TSP/ETP niches and/or a decline in pre‐thymic lymphoid progenitors. First, we used a multicongenic progenitor transfer approach to quantify the number of functional TSP niches from the onset of involution through middle‐age (12MO) (Ziętara et al., 2015). These studies revealed that the number of TSP niches is sustained through 12MO of age, despite substantial involution. We thus considered whether the reduction in ETP cellularity could be due to a reduced number of hematopoietic progenitors that seed the thymus. Surprisingly, we observe a significant decline in the number of lymphoid progenitors with T‐lineage potential in the BM and blood at 3MO of age, although their cell‐intrinsic ability to seed and differentiate in the thymus remains intact. Because Notch signaling plays a key role in promoting T‐lineage specification and progenitor differentiation, we also interrogated niche function by evaluating *Dll4* expression by cTECs and assessing Notch signaling activity in ETPs and BM progenitors. Notably, we find reduced Notch signaling in T‐lineage precursors in both the BM and thymus by 3MO of age. Collectively, these findings demonstrate a surprisingly early decline in BM lymphoid progenitors and circulating TSPs, along with diminished Notch signaling in the BM and thymus microenvironments, implicating both pre‐thymic and thymic changes in the early decline of ETP cellularity at the outset of thymus involution.

## 2 RESULTS

### 2.1 ETP and total thymocyte cellularity decline by 2MO of age

**FIGURE 1:**
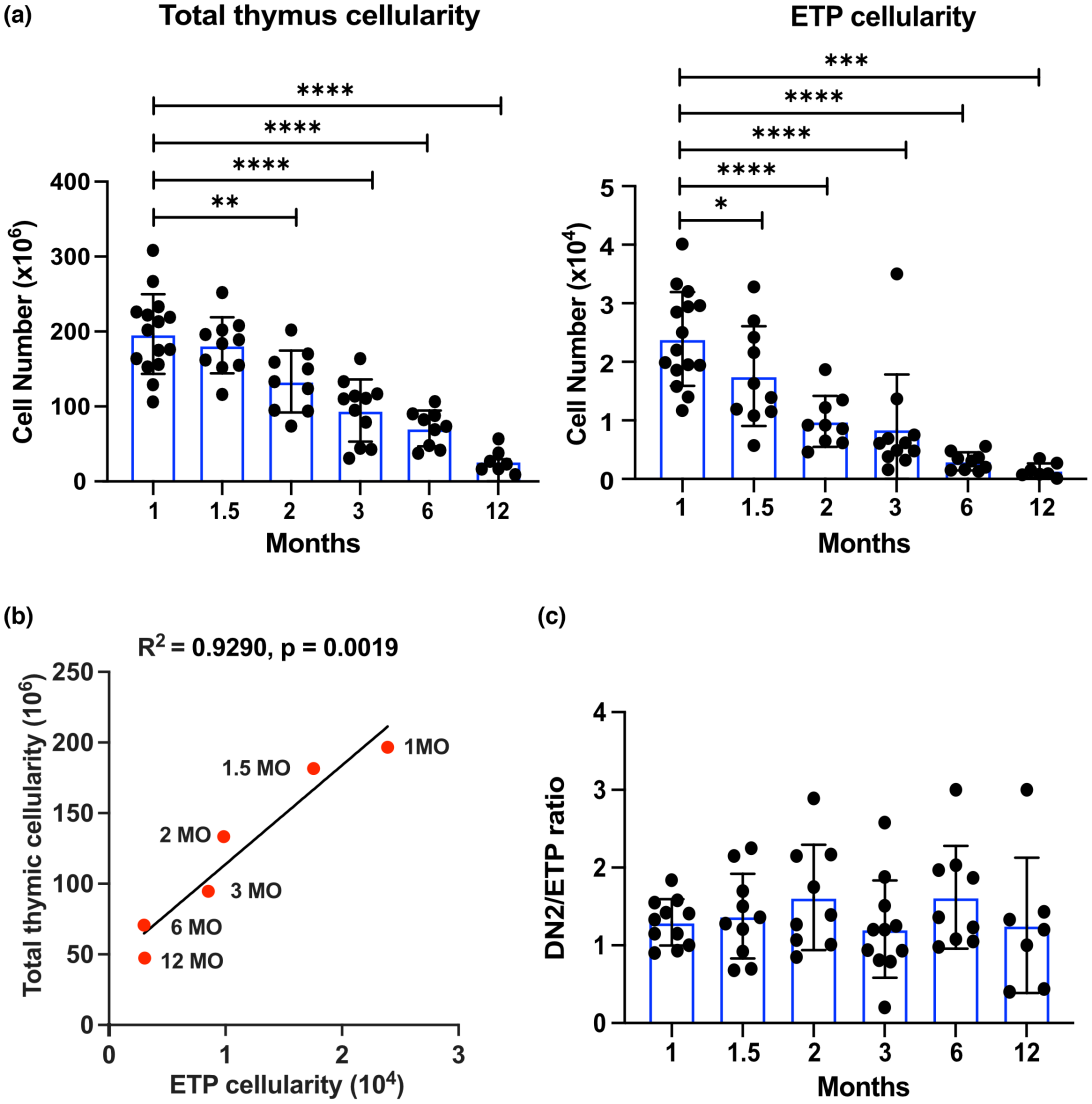
Early T‐cell progenitor (ETP) and total thymocyte cellularity declines by 2MO of age. (a) Quantification of total thymocytes and ETPs in C57BL/6J mice at the indicated ages. (b) Linear regression analysis of total thymic cellularity and ETP cellularity at each age. *R* ^2^ = coefficient of correlation (c) Ratio of DN2 to ETP cellularity at each age. Symbols represent (a, c) data from individual mice and (b) an average of 7–14 mice at each age. Bars represent means ± SEM from 3–4 independent experiments for each age group. Statistical analysis in (a) was performed using one‐way ANOVA with Dunnett's multiple comparisons test where *p < 0.05, **p < 0.01, ***p < 0.001, ****p < 0.0001.

We first quantified age‐associated changes in ETPs and downstream thymocyte subsets between 1 and 12MO of age (Figure 1 and Figure S1A). Total thymus cellularity declines with age, dropping significantly by 2MO, consistent with previous reports that thymus involution begins early in life (Figure 1a; Chen et al., 2009; Hale et al., 2006; Lepletier et al., 2019). Notably, ETP cellularity also declines progressively, starting at 1.5MO, which precedes, but otherwise mirrors the reduction in total thymocytes (Figure 1a). There is a strong correlation between ETP and total thymocyte numbers through 12MO of age (Figure 1b), and this correlation extends to subsequent thymocyte subsets (Figure S1B), showing that increased numbers of downstream thymocytes do not compensate for the loss of ETPs with age. Moreover, the consistent DN2 to ETP ratio over the first year of life further suggests that ETP differentiation is not impaired with age (Figure 1c).

### 2.2 The number of available thymic niches for TSPs does not decrease with age

**FIGURE 2:**
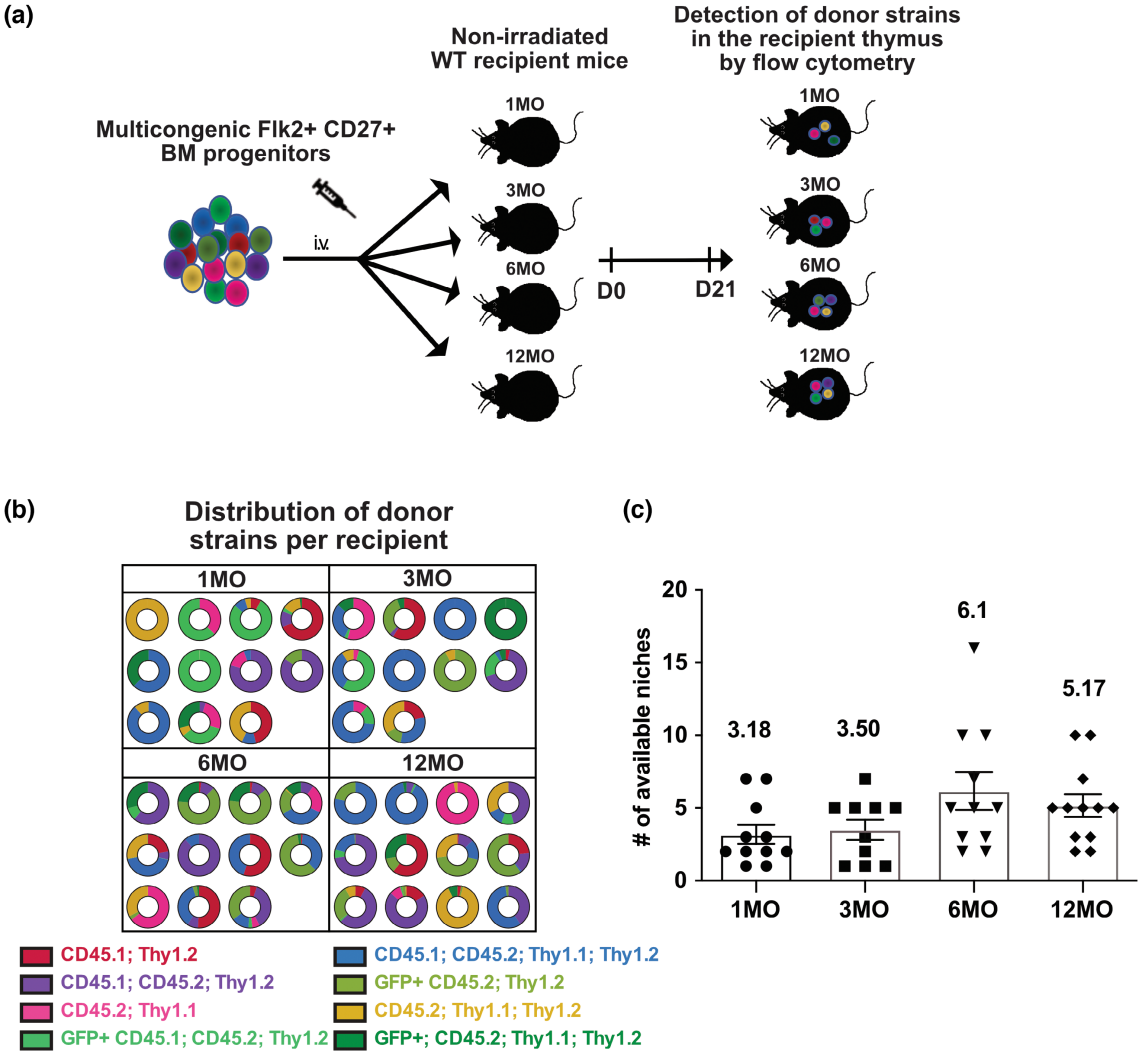
Number of available, functional TSP niches does not decline with age. (a) Schematic of the multicongenic barcoding experiment to quantify available niches in mice of different ages. Lin^−^Flk2^+^CD27^+^ BM progenitors were isolated from eight different congenic strains (Figure S2B), mixed at equal ratios and i.v. injected into non‐irradiated 1MO, 3MO, 6MO, and 12MO mice. After 21 days, the thymi from recipient mice were analyzed by flow cytometry to quantify the number of detectable donor strains that underwent T‐cell differentiation. (b) Pie charts illustrate the number and relative frequency of distinct color‐coded donor strains in individual recipients of the indicated ages. (c) The estimated number of available functional niches in each age recipient as determined by multinomial sampling. Symbols indicate data from individual mice; bars represent means ± SEM compiled from four independent experiments (*n* = 10–12 mice per recipient age).

The age‐associated decline in ETP cellularity could be a function of changes in the thymic stromal microenvironment and/or defects in pre‐thymic lymphoid progenitors. Given the important role of the thymic microenvironment in supporting all stages of developing T cells and its known impact on involution, we first tested whether the reduction in ETPs is due to an age‐related decline in the number of functional progenitor niches that support the early stages of thymopoiesis. We used a modification of a previously reported multicongenic progenitor transfer approach to quantify available, functional TSP niches from 1MO to 12MO of age. This approach previously revealed that a young thymus contains ~10 available niches that can be colonized by thymocyte progenitors at any one time (Ziętara et al., 2015). We FACS sorted Lin^−^Flk2^+^CD27^+^ BM progenitors, which encompass all thymus seeding and T‐cell progenitor activity in the BM (Serwold et al., 2009), from eight different congenic strains, mixed them at equal ratios, and transplanted them into non‐irradiated recipient mice of different ages (Figure 2a and Figure S2A,B). Recipient thymuses were analyzed after 21 days to allow sufficient time for donor progenitors to complete one wave of T‐cell differentiation (Serwold et al., 2009). We then quantified how many donor strains were present in each recipient thymus by flow cytometry (Figure S2C) and used mathematical modeling to estimate niche numbers. If the number of available niches that support TSP seeding declines with age, then fewer donor strains would be detected in thymuses of older recipients. However, the data did not show a decline in the number of available, functional TSP niches with age; instead, there was a trend toward increased niche availability in 6‐ and 12‐MO recipients (Figure 2b,c). Therefore, the age‐associated decline in ETP cellularity is not due to fewer functional niches available to TSPs.

### 2.3 1MO and 3MO T‐cell progenitors seed and thrive comparably in the thymus environment

**FIGURE 3:**
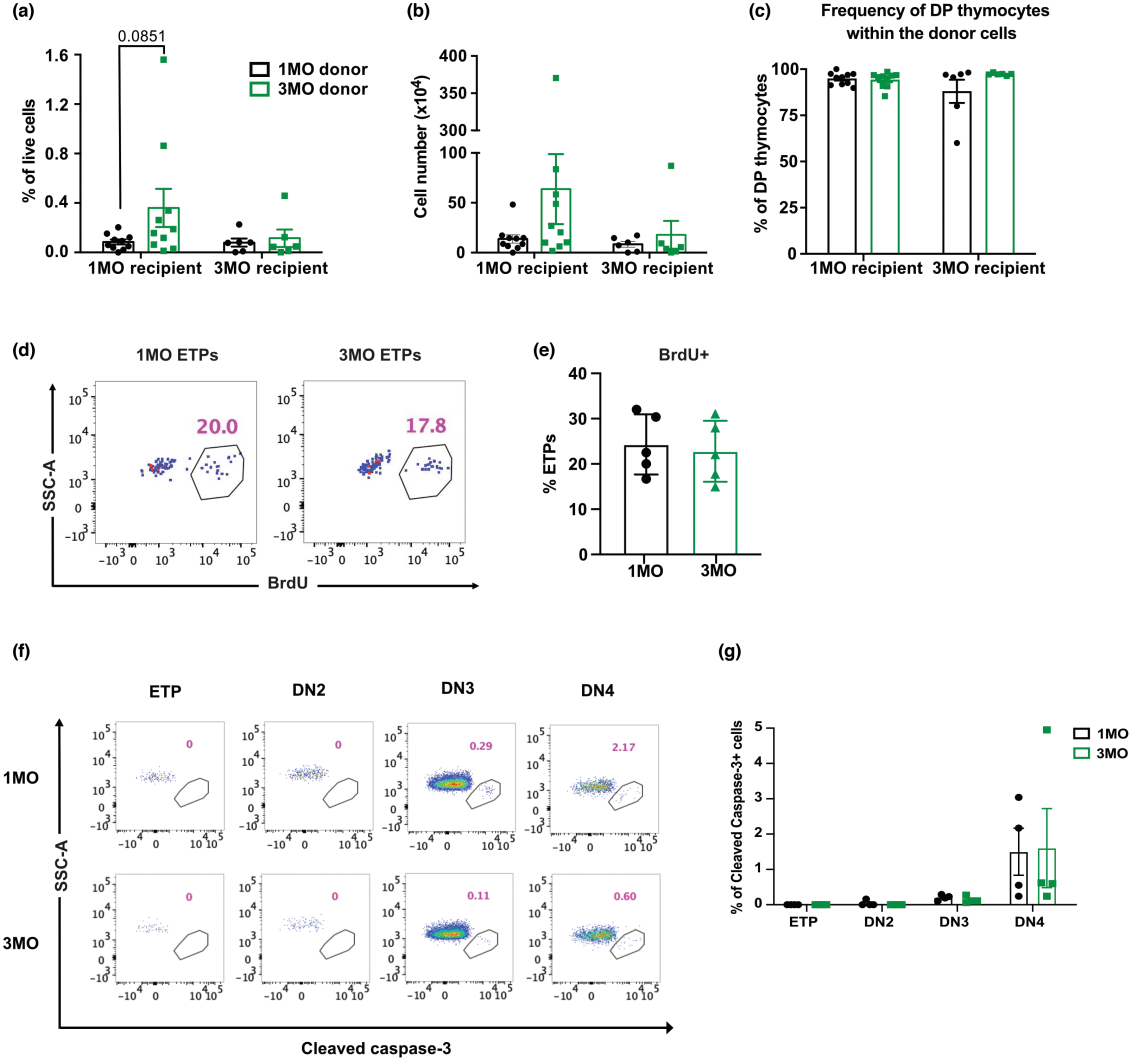
3MO T‐cell progenitors show comparable seeding and differentiation capacity relative to 1MO progenitors. (a–c) Donor chimerism in 1MO and 3MO recipient thymuses 21d after transplantation of an equal number of 1MO and 3MO Lin^−^Flk2^+^CD27^+^ BM progenitors into 1MO or 3MO non‐irradiated recipients. Quantification of the (a) frequency and (b) number of 1MO and 3MO donor‐derived thymocytes in recipients of the indicated ages. (c) Quantification of the percentage of DP thymocytes derived from 1MO and 3MO donors detected in recipient mice of the indicated ages. Data are pooled from four independent experiments (*n* = 6–10 mice per recipient age). (d) Representative flow cytometry plots and (e) quantification of the percentage of ETPs that incorporated bromodeoxyuridine (BrdU) 8 h after i.p. injection in mice of the indicated ages. Data are compiled from four independent experiments (*n* = 5 mice per age group). (f) Representative flow cytometry plots and (g) quantification of the frequency of cleaved caspase‐3^+^ cells in the indicated DN thymocyte subsets from 1MO and 3MO mice. Data are pooled from four independent experiments (*n* = 4 mice per age group). (a–c, e, g) Symbols represent data from individual mice. Bars represent means ± SEM. *p* value for (a) was obtained by two‐way ANOVA with Sidak's multiple comparison test.

We next asked whether the decline in ETPs reflects intrinsic defects in 3MO TSPs, compromising their ability to seed and differentiate in available thymic niches. Thus, we performed competitive heterochronic progenitor transfer assays in which isolated Lin^−^Flk2^+^CD27^+^ BM progenitors, obtained from 1MO and 3MO congenic donor strains, were mixed at an equal ratio and injected i.v. into 1MO versus 3MO non‐irradiated recipients. If BM progenitors from 3MO donors have intrinsic defects, then we would expect fewer 3MO compared to 1MO donor‐derived cells in recipient thymuses. However, comparable percentages and numbers of 1MO and 3MO donor cells were recovered from each thymus, in both 1MO and 3MO recipients (Figure 3a,b). These data indicate that TSPs from 3MO donors are fully competent to seed and thrive in the thymus. Moreover, there is no apparent defect in the differentiation capacity of 3MO TSPs, as a similar percentage of DP thymocytes are generated from 1MO versus 3MO donor cells (Figure 3c).

We next considered the possibility that changes in proliferation or survival could account for the decline in ETPs at 3MO. However, a comparable percentage of 1MO and 3MO ETPs incorporated BrdU (Figure 3d,e), and analysis of intracellular cleaved caspase‐3 showed that apoptosis is negligible in ETPs at both 1MO and 3MO of age (Figure 3f,g). Taken together, the data suggest that the decline in ETP numbers at 3MO of age is not a consequence of defects in the ability of 3MO TSPs to thrive in the thymus.

### 2.4 Lymphoid progenitors with T‐lineage potential decline in the BM and blood of 3MO mice

**FIGURE 4:**
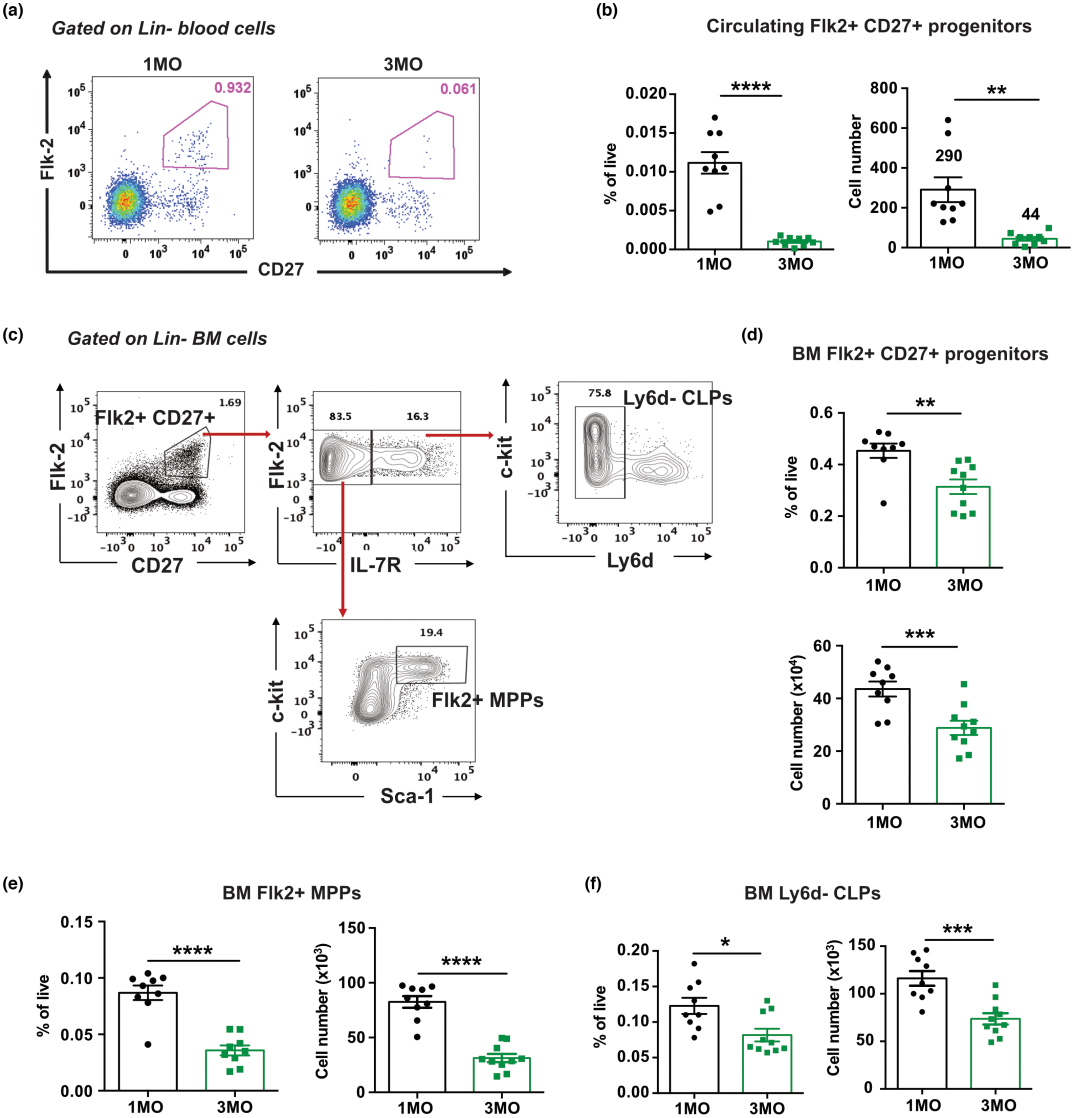
The number of circulating TSPs and BM lymphoid progenitors declines between 1MO and 3MO of age. (a) Representative flow cytometry plots showing the percentage of Flk2^+^CD27^+^ progenitors within the Lin^−^ compartment of the blood in 1MO and 3MO mice. (b) Frequency and absolute cell numbers of circulating lymphoid progenitors in total blood of 1MO and 3MO mice. (c) Representative gating strategy for quantification of Lin^−^Flk2^+^CD27^+^ progenitors, Ly6d^−^ common lymphoid progenitors (CLPs), and Flk2^+^ multipotent progenitors (MPPs) in the BM. (d–f) Frequency and absolute numbers of (d) Flk2^+^CD27^+^ progenitors, (e) Flk2^+^ MPPs and (f) Ly6d^−^ CLPs in BM of 1MO and 3MO mice. Symbols represent data from individual mice at each age. Bars represent means ± SEM of data compiled from three independent experiments (*n* = 9 mice per age group). Statistical analysis was performed using Student *t* test, *p < 0.05, **p < 0.01, ***p < 0.001, ****p < 0.0001.

As we found no apparent defects in either the availability of functional TSP niches with age or the ability of 3MO TSPs to enter and thrive in those niches, we asked whether the number of BM or circulating lymphoid progenitors decreases by 3MO of age. A significant reduction in the number of TSPs could play a key role in the age‐associated decline in ETP cellularity. To assess this possibility, we used flow cytometry to quantify the total number of circulating Lin^−^Flk2^+^CD27^+^ cells, which contain all thymic seeding activity (Serwold et al., 2009), in the blood of each mouse (Figure 4a). We also quantified upstream Lin^−^Flk2^+^CD27^+^ lymphoid progenitors in the BM (Figure 4c). Interestingly, the frequency and number of Flk2^+^ CD27^+^ lymphoid progenitors in both blood and BM undergo a significant decline between 1MO and 3MO of age (Figure 4b,d). Flk2^+^ MPPs and Ly6d^−^ CLPs (Inlay et al., 2009) are the lymphoid progenitors with T‐lineage potential within the Lin^−^Flk2^+^CD27^+^ compartment (Serwold et al., 2009), and both of these subsets also decline significantly by 3MO of age (Figure 4e,f). These results demonstrate a surprisingly early reduction in the number of BM lymphoid progenitors and circulating TSPs, indicating that the decrease in ETP cellularity by 3MO is likely due, at least in part, to a reduction in the number of progenitors available to enter the thymus and differentiate therein.

### 2.5 Notch signaling declines in BM lymphoid progenitors by 3MO

**FIGURE 5:**
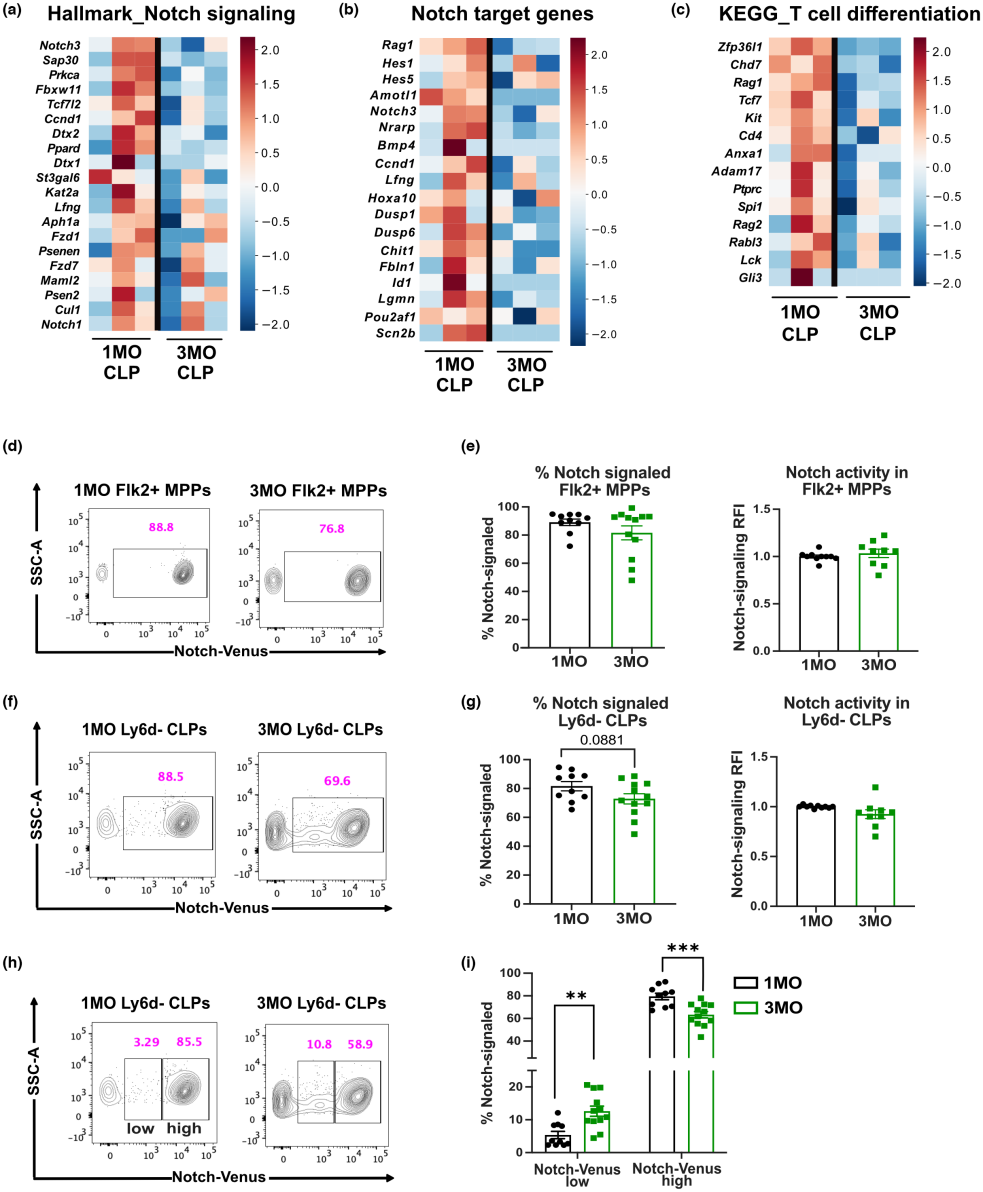
Age‐associated changes in Notch signaling accompany the decline in BM lymphoid progenitors at 3MO. (a–c) Heatmaps show row‐normalized z‐scores of gene expression values indicating enrichment of (a) the Notch signaling pathway (Hallmark), (b) Notch target genes, and (c) the T‐cell differentiation (KEGG) pathway in 1MO versus 3MO Ly6d^−^ CLPs (*n* = 3 independent experiments). (d–g) Representative flow cytometry plots, as well as quantification of the frequencies and relative fluorescence intensities (RFIs) of Notch‐Venus reporter expression in (d, e) BM Flk2^+^ MPPs and (f, g) BM Ly6d^−^ CLPs. (h) Representative flow cytometry plots showing gating to distinguish Notch‐Venus low from Notch‐Venus high cells. (i) Frequencies of Notch‐Venus low and Notch‐Venus high Ly6d^−^ CLPs in 1MO and 3MO BM. (d–i) Data are pooled from five independent experiments (*n* = 9–12 mice per age group).

To identify age‐associated changes that could contribute to the reduction in Ly6d^−^ CLP and Flk2^+^ MPP by 3MO, we carried out transcriptional profiling of these progenitors at 1MO versus 3MO of age. Gene set enrichment analysis (Subramanian et al., 2005) revealed age‐associated changes in expression of genes and related pathways in each BM progenitor subset, with more significant differences observed for Ly6d^−^ CLPs relative to Flk2^+^ MPPs (Figure 5a–c and Figures S3 and S4). Of note, enrichment of the Notch signaling pathway declines between 1 and 3MO of age in Ly6d^−^ CLPs, but not in Flk2^+^ MPP (Figure 5a and Figure S4A).

Notch signaling in BM lymphoid progenitors plays a critical role in differentiation of T‐lineage progenitors (Chen et al., 2019; Tikhonova et al., 2019; Yu et al., 2015); thus, we considered the possibility that reduced Notch signaling could contribute to the decrease in BM lymphoid progenitors by 3MO of age. To further test whether Notch signaling declines, we evaluated expression of known Notch target genes in our datasets (Dong et al., 2021; Wang et al., 2014; Weerkamp, Luis, et al., 2006), and found reduced expression in 3MO versus 1MO Ly6d^−^ CLPs, but not in Flk2^+^ MPPs (Figure 5b and Figure S4B). Interestingly, expression of genes associated with T‐cell differentiation also declines in Ly6d^−^ CLPs, but not in Flk2^+^ MPPs, by 3MO of age, including known Notch targets (i.e., *Rag1* and *Rag2*; Figure 5c and Figure S4C). Altogether, these data implicate diminished Notch signaling as a potential mechanism contributing to the reduced number of T‐lineage progenitors in the BM by 3MO of age.

To further test this hypothesis, we used the CBF‐H2B‐Venus strain in which expression of an H2B‐Venus fusion protein is driven by Notch activation (Nowotschin et al., 2013). Due to perdurance of the H2B‐Venus fluorescent signal, this reporter identifies cells that are currently undergoing or have recently undergone Notch signaling. Although the frequency of Notch‐signaled Flk2^+^ MPPs and Ly6d^−^ CLPs did not decline significantly between 1MO and 3MO (Figure 5d–g), there was a notable enrichment of Venus reporter low Ly6d^−^ CLPs at 3MO relative to 1MO (Figure 5h,i). The decline in the Notch signaling reporter activity in 3MO Ly6d^−^ CLPs indicates that Notch signaling wanes at an early age in BM progenitors that contain T‐lineage potential.

Analysis of transcriptional profiling data revealed age‐associated changes in other pathways that could also impact T‐cell progenitors in the BM. Expression of genes in the Wnt‐β‐catenin pathway declines by 3MO in Ly6d^−^ CLPs, but not in Flk2^+^ MPPs (Figures S3A,B and S4D), and Wnt signaling has been implicated in regulating multiple steps of hematopoiesis and lymphopoiesis (Luis et al., 2012; Weerkamp, Baert, et al., 2006). Additionally, genes associated with ECM‐receptor interactions decline in 3MO Ly6d^−^ CLPs, but not in Flk2^+^ MPPs, potentially reducing interactions between lymphoid progenitors and supportive BM niches (Figures S3A,B and S4E). Furthermore, the expression of genes, including integrins and adhesion molecules, associated with the leukocyte‐transendothelial migration pathway declines by 3MO in Ly6d^−^ CLPs, but not in Flk2^+^MPPs (Figures S3A,B and S4F), which could impair egress of TSPs from the BM. Collectively, these findings implicate early changes in Notch signaling and other pathways involved in regulation of lymphopoiesis and cellular interactions in the reduction in T‐lineage progenitors in the BM.

### 2.6 Notch ligand expression in thymic stromal cells and Notch signaling in ETPs are diminished by 3MO

**FIGURE 6:**
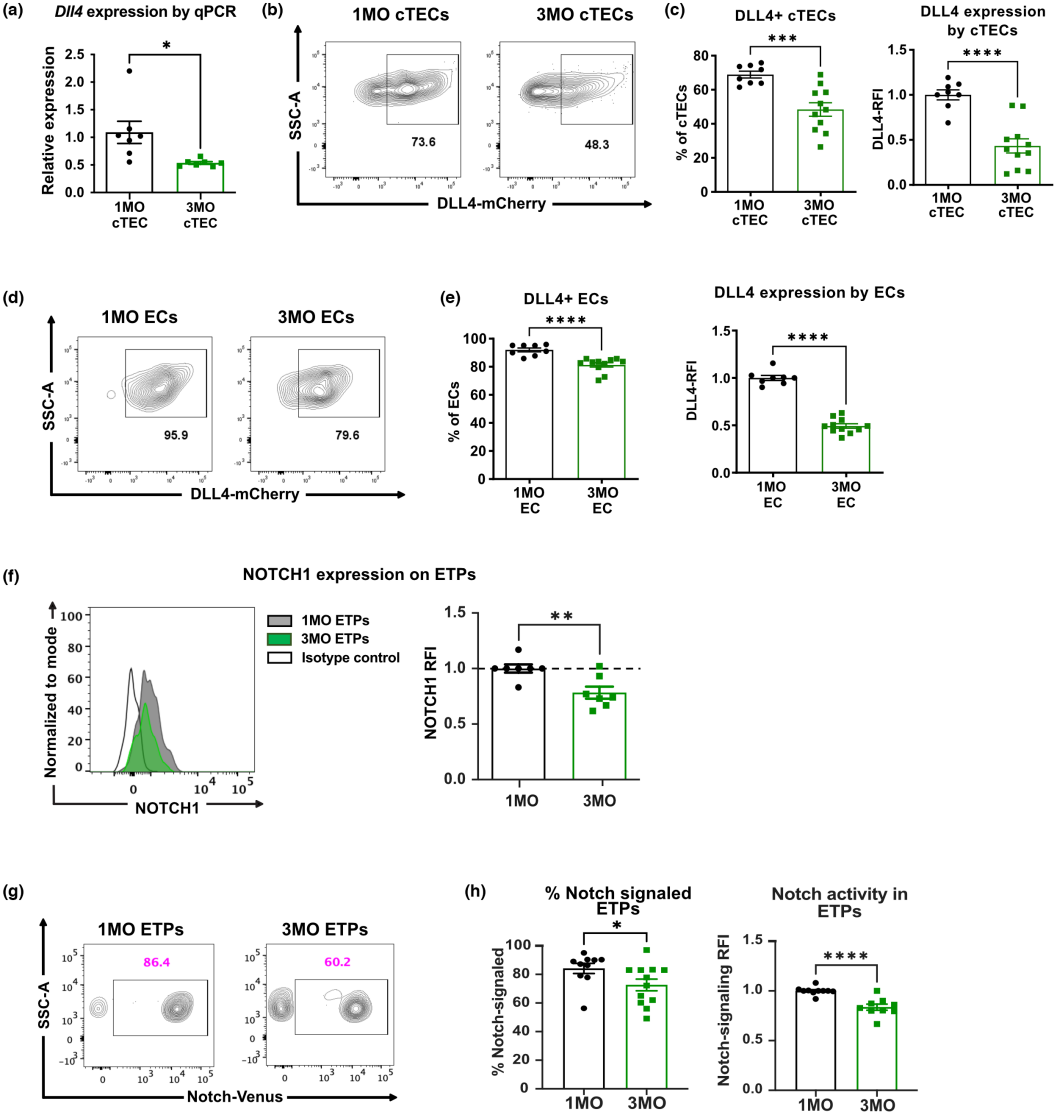
Age‐associated changes in Notch ligand expression and Notch signaling in the thymus correlate with the decline in ETPs at 3MO. (a) qPCR analysis showing relative *Dll4* expression by CD45^−^ EpCAM^+^ Ly51^+^ cTECs from 1MO and 3MO C57BL/6J mice. Data are pooled from two biological experiments with 3–4 technical replicates per experiment, and expression levels were normalized to those at 1MO of age. (b, d) Representative flow cytometry plots and (c, e) quantification of the frequency of *Dll4*‐reporter^+^ cells and *Dll4*‐mCherry RFIs in (b, c) CD45^−^ EpCAM^+^ Ly51^+^ cTECs, and (d, e) CD45^−^ CD31^+^ endothelial cells (ECs) from *Dll4‐mCherry* reporter mice. Data are normalized to the average *Dll4‐mCherry* expression by (c) 1MO cTECs and (e) 1MO ECs. (c, e) Data are pooled from four independent experiments (*n* = 8–10 mice). (f) Representative histogram (left) and quantification of RFIs (right) of NOTCH1 cell surface expression on thymic ETPs. Data are normalized to the average NOTCH1 MFI of 1MO mice in each individual experiment. Data are pooled from three independent experiments (*n* = 7 mice per age group). (g) Representative flow cytometry plots and (h) frequencies and RFIs of Notch‐Venus reporter expression in thymic ETPs. Data are pooled from five independent experiments (*n* = 9–12 mice per age group). (c, e, f, h) Symbols represent data from individual mice at each age and bars represent means ± SEM. Statistical analysis was performed using Student *t* test, *p < 0.05, **p < 0.01, ***p < 0.001, ****p < 0.0001.

Activation of the Notch signaling pathway in the thymus is also essential for ETP T‐cell lineage commitment and subsequent T‐cell development (Han & Zúñiga‐Pflücker, 2021), and the Notch ligand *Dll4* is expressed by both cTECs and endothelial cells (ECs) in the thymus (Buono et al., 2016; Hozumi, Mailhos, et al., 2008; Velardi et al., 2014). Our previously reported gene expression data show downregulation of *Dll4* in cTECs between 1 and 3MO of age (Ki et al., 2014). Quantitative RT‐PCR analysis of isolated 1MO and 3MO cTECs confirms reduced *Dll4* expression by 3MO of age (Figure 6a). To validate the early age‐associated decline in *Dll4* expression in the thymus environment, we analyzed cTECs and thymic ECs from *Dll4* reporter mice (Tikhonova et al., 2019). Interestingly, *Dll4* expression declines in both cTECs and ECs between 1MO and 3MO of age (Figure 6b–e). In addition, the level of NOTCH1 expression on ETPs declines significantly by 3MO (Figure 6f,h), consistent with the fact that *Notch1* is a target gene of NOTCH signaling (Wang et al., 2014; Weerkamp, Luis, et al., 2006). To determine whether decreased levels of NOTCH1 on ETPs and reduced expression *of Dll4* by cTECs and ECs at 3MO of age are associated with diminished Notch signaling in ETPs, we first tested whether the Notch‐Venus reporter is faithfully expressed in immature thymocyte subsets known to undergo Notch signaling. As expected, reporter levels were high in ETPs and DN2s and dropped in the DN3 and DN4 subsets (Figure S5). Notably, the frequency of ETPs undergoing Notch signaling declines between 1MO and 3MO of age (Figure 6g,h). Also, the lower relative mean fluorescence intensity (RFI) of the Venus reporter in 3MO ETPs indicates a reduced level of Notch signaling. Taken together, these data show that the early decline in ETP cellularity is associated with diminished Notch signaling activity in T‐lineage progenitors in both the BM and thymus microenvironments.

## 3 DISCUSSION

It is well established that thymus cellularity and T‐cell output decline during age‐associated thymus involution (Chinn et al., 2012). While prior studies focused on comparative analysis of thymuses from young mice (1–4MO of age) versus severely involuted thymuses from middle‐aged and old mice (9–28MO of age), the present investigation focuses on changes that occur during the initial stages of thymus involution between 1 and 3MO of age. In agreement with other reports (Chen et al., 2009; Hale et al., 2006; Lepletier et al., 2019), we find a significant decline in thymus cellularity by 3MO of age. Furthermore, the progressive reduction in total thymocyte numbers with age is highly correlated with a reduction in the number of ETPs, which contain progenitors that give rise to all subsequent stages of developing T cells. These results suggest that the early decline in ETPs plays a key role in initiating age‐associated thymus atrophy. Given that TSPs, ETPs and all subsequent stages of developing T cells rely on inductive and supportive signals from the thymic microenvironment, which undergoes profound age‐associated changes during thymus involution, we first tested whether a reduction in the thymic niches that support TSPs and ETPs are diminished by 3MO of age. Unexpectedly, we did not find an age‐related decline in the number of functional, available TSP niches between 1 and 12MO of age. In fact, there was a slight increase in these niches by 12MO of age. Thus, we subsequently investigated whether the early decline in ETP numbers reflects a reduction in BM and/or circulating T‐cell progenitors. Our studies reveal a dramatic decline in circulating Flk2^+^CD27^+^ progenitors, which contain TSPs, as well as a reduction in BM lymphoid progenitors by 3MO of age. We provide multiple lines of evidence implicating an early age‐associated decline in Notch signaling as a factor contributing to the decreased cellularity of T‐lineage progenitors in the BM. Furthermore, expression of the Notch ligand DLL4 by cTECs and thymic ECs is reduced by 3MO, as is Notch signaling activity in ETPs. Overall, our results suggest that both a decline in pre‐thymic lymphoid progenitors in the BM and blood, along with changes in the BM and thymic stromal microenvironments, contribute to the early onset of thymus involution by 3MO of age.

It has previously been established that hematopoietic progenitors in the BM become myeloid‐biased with age. For example, transplantation experiments revealed that HSCs from aged mice are biased toward a myeloid fate (Beerman et al., 2010; Busch et al., 2015; Min et al., 2004; Rossi, Corbel, et al., 2005; Young et al., 2016). Moreover, fate mapping studies have shown that MPPs from old mice transit more efficiently into common myeloid progenitors (CMPs) than CLPs (Busch et al., 2015). Nevertheless, the differentiation potential of lymphoid‐biased HSCs on a per cell basis does not decline with age (Dorshkind et al., 2020; Montecino‐Rodriguez et al., 2019). These prior studies assessed the decline in BM lymphoid potential between young (1–4MO) and much older mice (10–28MO). Since we find that ETP numbers decline by 3MO of age, we explored the possibility that a reduction in BM lymphoid progenitors contributes to the early decline in ETPs. Importantly, we found a striking decrease in the number of circulating TSPs and the number and percentage of the BM progenitors Ly6d^−^ CLPs and Flk2^+^ MPPs at 3MO. Thus, diminished production and/or egress of T‐lineage progenitors from the BM likely contributes to the early reduction in ETPs. Interestingly, a fetal‐derived lymphoid‐biased HSC subset persists in the BM until at least 2 weeks of age before being lost in adult mice (Beaudin et al., 2016). Also, embryonic‐derived hematopoietic progenitors contribute to thymopoiesis through 7 weeks of age (Montecino‐Rodriguez et al., 2019). These studies raise the possibility that the early decline in BM lymphoid progenitors and ETPs could reflect a reduced contribution of embryonically derived hematopoietic progenitors. However, competitive heterochronic progenitor transfer assays showed no cell‐intrinsic defect in the ability of 3MO Flk2^+^CD27^+^ lymphoid progenitors to seed the thymus or differentiate therein, indicating that even if the switch from fetal to adult hematopoiesis results in fewer TSPs, they are not less functional on a per cell basis. To our knowledge, the present study is the first to show a substantial reduction in BM lymphoid progenitors and circulating TSPs by 3MO of age.

As lymphoid progenitor activity is regulated by niche factors in the BM and thymus microenvironments, age‐associated changes in stromal cells could play a role in the early decline of BM lymphoid progenitors and ETPs. Several observations in the present report suggest that diminished Notch signaling contributes to the early decline in BM lymphoid progenitors and ETPs between 1MO and 3MO of age. Transcriptional profiling revealed a decline in the Notch signaling pathway and expression of Notch target genes in Ly6d^−^ CLPs between 1 and 3 MO of age. Consistent with these findings, analysis of Notch signaling using the CBF‐H2B‐Venus Notch reporter strain (Nowotschin et al., 2013) demonstrated an age‐related decline in Notch signaling in BM‐resident CLPs. We note that the decline in expression of genes associated with Notch signaling in Ly6d^−^CLPs, but not in Flk2^+^ MPPs, is consistent with the reduction in Notch signaling reporter levels in CLPs, but not in MPPs, by 3‐MO of age. Notably, Notch signaling in the BM microenvironment is essential to sustain a normal number of Ly6d^−^ CLPs and downstream thymic ETPs, although it is not needed to sustain LMPPs (Tikhonova et al., 2019; Yu et al., 2015). Thus, the observed reduction in Notch signaling in Ly6d^−^ CLPs by 3MO is likely to impair production of T‐lineage progenitors. Transcriptional profiling studies also demonstrate that by 3MO of age Ly6d^−^ CLPs have reduced expression of genes like integrins and adhesion molecules that could both impair their interactions with a supportive BM stromal niche and reduce egress of T‐lineage progenitors into circulation. However, *Cxcr4* expression did not increase in 3MO relative to 1MO Ly6d^−^ CLPs (not shown), indicating that CXCR4‐mediated retention of T‐lineage progenitors in the BM is not likely responsible for the reduction in circulating TSPs.

Heterochronic BM transplantation experiments showed that successful T‐cell reconstitution depends on the age of the recipient thymus rather than on the age of donor hematopoietic progenitors (Mackall et al., 1998; Zhu et al., 2007), consistent with the notion that thymic stromal support of thymopoiesis wanes with age. Alterations in the composition, organization, and/or function of the thymus microenvironment could impair seeding and/or differentiation of TSPs, which enter the thymus on a periodic basis to occupy a limited number of stromal cell niches (Donskoy & Goldschneider, 1992; Foss et al., 2001; Ziętara et al., 2015). Therefore, we asked whether the availability of functional TSP niches diminishes with age. Using multicongenic barcoding progenitor transfers, combined with mathematical modeling, thymuses from 1MO and 3MO old mice were found to contain a comparable number of available TSP niches. Thus, the early decline in ETP numbers is not a function of reduced TSP niche availability. To the contrary, our results showed an upward trend in the number of available TSP niches with age. Previous investigations have reported evidence for a feedback loop in which key TSP niche factors and T‐cell progenitor entry are restricted by the number of intrathymic T‐cell precursors (Prockop & Petrie, 2004; Rossi, Bryder, et al., 2005; Ziętara et al., 2015). Since we find a substantial reduction in the number of circulating TSPs and intrathymic ETPs as early as 3MO of age, it is possible that the observed increase in available TSP niches with advancing age is due, at least in part, to a decline in feedback from TSPs and/or ETPs.

As TSP transition into the thymus, stromal cells like ECs and cTECs play a critical role in supporting their survival, proliferation, and T‐lineage commitment by providing niche factors, like DLL4, KITL, and IL‐7 (Buono et al., 2016; Han & Zúñiga‐Pflücker, 2021; Krueger et al., 2017; Lancaster et al., 2018). Given that the TEC compartment deteriorates during thymus involution, we considered the possibility that ETP niche factors become diminished with age. It is well established that Notch signaling plays an essential role in thymic T‐lymphopoiesis (Chen et al., 2009; Han & Zúñiga‐Pflücker, 2021; Hozumi, Mailhos, et al., 2008; Koch et al., 2008; Wilson et al., 2001). NOTCH1 is expressed by ETPs, while the critical Notch ligand DLL4 is expressed by thymic ECs and cTECs (Buono et al., 2016; Han & Zúñiga‐Pflücker, 2021; Velardi et al., 2014). We found that Notch expression and signaling are reduced in ETPs by 3MO of age, consistent with diminished *Dll4* expression in both cTECs and ECs. Importantly, Notch impacts T‐cell development in a dose‐dependent manner, such that lymphoid progenitors with heterozygous Notch deficiency are at a competitive disadvantage in their ability to generate ETPs and downstream thymocytes (Tan et al., 2005). Also, even a partial reduction in *Dll4* expression by TECs results in fewer ETPs and downstream thymocytes (Velardi et al., 2014). Thus, while the number of available TSP niches does not decline by 3MO, the reduced expression of *Dll4* by thymic stromal cells, together with a reduction in Notch signaling reporter levels in ETPs, strongly indicates that the thymus environment has a reduced capacity to support Notch signaling in T‐lineage progenitors, contributing to the early decline in ETPs and downstream progeny.

Collectively, our results indicate that an early age‐associated decline in Notch signaling in T‐cell progenitors results in a reduced number of BM lymphoid progenitors, circulating TSPs and thymic ETPs at the outset of thymus involution. This link will be further evaluated in future studies. In summary, this study shows that reduced BM lymphoid potential and alterations in thymic stromal cells during the first 3MO of life synergize to initiate the earliest stages of thymus involution.

### 3.1 Experimental procedures

#### 3.1.1 Mice

C57BL/6J (CD45.2, Thy1.2), B6.PL‐Thy1a/CyJ 9 (CD45.2, Thy1.1), B6.SJL‐*Ptprc* ^*a*^ *Pepc* ^*b*^/BoyJ (CD45.1, Thy1.2), C57BL/6‐Tg(CAG‐EGFP)1Osb/J (GFP) (Okabe et al., 1997), and Tg(Cp‐HIST1H2BB/Venus)47Hadj/J (Notch‐Venus reporter) (Nowotschin et al., 2013) mice were obtained from Jackson Laboratories and bred in‐house. F1 mice for multicongenic barcoding experiments were bred in‐house from CD45 congenic, Thy1 congenic, and EGFP strains (Figure S2B). *Dll4‐mCherry* mice were generously provided by Dr. Iannis Aifantis (NYU School of Medicine, NY) (Tikhonova et al., 2019). C57BL/6J recipient mice for multicongenic barcoding experiments were obtained from the National Institute of Aging. All mice were maintained under specific pathogen‐free conditions at the animal facilities at the University of Texas at Austin and the University of Texas MD Anderson Cancer Center, Smithville. All experimental procedures were performed in accordance with the Institutional Animal Care and Use Committees.

### 3.2 Multicongenic barcoding progenitor transfer assay

Bone marrow cells were obtained from the long bones (femur, tibia, humerus, radius, and ulna) of 4‐ to 6‐week‐old mice of eight different congenic backgrounds (Figure S2B). Bones were crushed and single‐cell suspensions were prepared in FACS wash buffer (PBS+ 2% FBS), followed by RBC lysis (RBC lysis buffer, BioLegend). Cells were stained with lineage (Lin)‐specific antibodies against CD11b, Gr‐1, Ter‐119, B220, CD19, CD3, and CD8 (all from BioXCell), and Lin^+^ cells were depleted with sheep anti‐rat IgG immunomagnetic beads (Dynabeads, Invitrogen). Cells were then immunostained, and Lin^−^Flk2^+^CD27^+^ BM progenitors from F1 congenic strains were FACS purified (Figure S2A). Progenitors from each of the eight congenic strains were mixed at equal ratios, and a total of 100,000 cells were retro‐orbitally injected into non‐irradiated 1MO, 3MO, 6MO, and 12MO C57BL/6J recipient mice. Thymocytes were analyzed after 21 days by flow cytometry to distinguish the contributions of distinct congenic donor strains. 2.5 million events were recorded per recipient thymus, and successful thymic colonization was scored if a minimum of 40 cells of a given donor strain was detected. Quantification of niches was performed using a multinomial sampling algorithm (see Bayesian estimation of TSP niche numbers).

### 3.3 Competitive heterochronic progenitor seeding assay

Lin^−^Flk2^+^CD27^+^ progenitors were isolated from the long bones of 1MO and 3MO congenic donor mice as described above. 1MO and 3MO progenitors were mixed at 1:1 ratio, and 100,000 cells were retro‐orbitally injected into 1MO and 3MO non‐irradiated C57BL/6J recipient mice. After 21 days, donor chimerism was analyzed in the recipient thymi by flow cytometry.

### 3.4 Flow cytometry

For analysis of thymocyte subsets, cell suspensions were prepared by manually dissociating thymuses in FACS wash buffer (FWB; PBS+ 2% bovine calf serum), and the cells were filtered through a 40 μM cell strainer (Fisher). For isolating cTECs and ECs, thymi were isolated and cleaned of fat and connective tissue and placed in PBS. Thymic lobes were cut into small pieces and enzymatically digested as previously described (Seach et al., 2012). Briefly, 0.2 mg of liberase TM (Sigma Aldrich) and 80 U of DNAse I (Sigma Aldrich) were used per thymus tissue for digestion. An enzymatic mixture containing Liberase TM and DNAse 1 in 2 mL PBS were added to the thymic lobes and gently agitated in a 37°C shaking incubator for 10 minutes for a total of four digestion steps. At each digestion step, supernatant was collected into a 50 mL tube containing FWB (PBS+ 2% bovine calf serum+5 mM EDTA). Cells were centrifuged at 1250 rpm for 5 min at 4°C, and the pellet was resuspended in 5 mL of FWB. Cells were depleted of thymocytes using anti‐mouse CD45 Microbeads (Miltenyi Biotech) on the autoMACS® Pro Separator according to manufacturer's recommendations. Enriched stromal cells were immunostained before FACS sorting cTECs.

For quantification of circulating Lin^−^Flk2^+^CD27^+^ progenitors, following euthanasia, blood was collected immediately by making an incision in the right atrium and puncturing the left ventricle with a syringe. All available blood from the mice was collected from the chest cavity by perfusing with 10 mM of ethylenediaminetetraacetic acid/phosphate‐buffered saline. RBCs were lysed with RBC lysis buffer (BioLegend) twice, and white‐blood cells were immunostained for flow cytometry. For quantification of BM lymphoid progenitors, long bones from each mouse were collected and crushed to obtain single‐cell suspensions, prior to filtering through a 40 μM cell strainer (Fisher) and lysing RBCs with RBC lysis buffer (BioLegend).

For preparation for flow cytometry, 6–10 million cells were immunostained with the fluorescently conjugated antibodies for 20 min on ice in FWB prior to washing in FWB and resuspending in FWB plus propidium iodide (Enzo) before flow‐cytometric analysis. For intracellular staining of BrdU and cleaved caspase‐3, cells were stained with antibodies for cell surface markers in FWB plus fixable viability dye (Zombie Red; BioLegend), then fixed and permeabilized using the BrdU APC kit (BD Biosciences) according to manufacturer's protocol. Then, the cells were stained with anti‐BrdU and anti‐cleaved caspase‐3 fluorescently conjugated antibodies. Flow cytometry data were acquired on a BD LSRFortessa™ or/and cells were FACS sorted on a BD FACSAria™ or BD FACSAria™ Fusion and analyzed using FlowJo software (V9.9.6 and V10.8.0, Tree Star Inc). A complete list of antibodies used for FACS analysis and sorting are provided in Table S1.

### 3.5 BrdU assay

Mice were weighed to determine the amount of BrdU to be injected per gram of body weight. 0.1 mg of BrdU per gram of body weight was injected intra‐peritoneally into 1MO and 3MO C57BL/6J mice. After 8 h, thymocytes were isolated to assess BrdU incorporation by flow cytometry.

### 3.6 cDNA preparation and quantitative PCR

FACS‐purified cTECs from 1MO and 3MO C57BL/6J mice were resuspended in TRIzol reagent (Invitrogen) to isolate RNA. cDNA was prepared using the qScript cDNA synthesis kit (Quantobio) according to manufacturer's instructions. qRT‐PCR experiments were performed on the Viia7 Real‐time PCR system (Thermo Fisher Scientific) using the following SYBR green and Taqman probes: DLL4 (SYBR green), actin (SYBR Green), and mouse alpha tubulin (Taqman). The primer sequences for DLL4 are as follows: forward primer, 5′‐AGGTGCCACTTCGGTTACAC‐3′; reverse primer, 5′‐GGGAGAGCAAATGGCTGATA‐3′. Gene expression levels were normalized relative to β‐actin.

### 3.7 Bayesian estimation of TSP niche numbers

For quantification of thymic niches, a Bayesian technique was implemented to estimate the number of niches present in recipient mice from the missing number of donors detected. Generalized linear models were used to test whether the distributions of the estimated number of niches present in each age category declines with increasing age. General linear modeling was performed with Poisson response and a log link function with age as the only categorical covariate. Test statistic (χ^2^) was used to test the trend of number of niches over age groups. For estimation, the donor probabilities from the proportion of donor cells injected in each mouse were calculated. A posterior likelihood was computed using sampling from a multinomial distribution where *L* is the number of iterations, *N* is the number of niches, and *k* is the number of distinct donors. This likelihood was then normalized to give a probability distribution for each likely value of missing donors. The maximum estimate of *N* that is drawn from the posterior distributions (MAP) was used as the most likely estimate of available niches. Batch to batch variation (i.e., replicate experiments) was used to test for potential bias due to batch effects. Regression analysis was used to assess whether there are differences in available niches by age.

### 3.8 Sample preparation for RNA‐seq

Lineage‐depleted BM cells were isolated from long bones of 1MO mice and 3MO mice, as described above. 50,000–100,000 Ly6d^−^ CLPs and Flk2^+^ MPPs were FACS‐purified per mouse (gating strategy as shown in Figure 4c). Total RNA was extracted using Direct‐zol RNA Microprep kit (Zymo Research) according to manufacturer's instructions. mRNA was isolated using the Poly(A) Purist‐MAG kit (ThermoFisher), according to manufacturer's instructions mRNA quality was assessed on an Agilent Bioanalyzer using the Agilent RNA 6000 Pico kit (Agilent). Libraries were prepared at the University of Texas Genomic Sequencing and Analysis Facility, according to manufacturer's instructions, using the NEBNext Ultra II Direction RNA kit (NEB, product number E7760). The resulting libraries were tagged with unique dual indices and checked for size and quality using the Agilent High Sensitivity DNA Kit (Agilent). Library concentrations were measured using the KAPA SYBR Fast qPCR kit and loaded for sequencing on the NovaSeq 6000 instrument (paired‐end 2X150, or single‐end, 100 cycles) with a target of 20 million reads per sample.

### 3.9 RNA‐seq data analysis

Data were processed using the nf‐core Nextflow pipeline (Ewels et al., 2020). The pipeline entails QC and merging of fastQ files with FastQC, UMI extraction with UMI‐tools (Smith et al., 2017), adapter and quality trimming with Trim Galore!, removal of ribosomal RNA with SortMeRNA (Kopylova et al., 2012), alignment with STAR v2.7.10b, transcript quantification with Salmon (Patro et al., 2017), and further quality control and normalization with DESeq2 (Love et al., 2014). Differential expression between 1MO and 3MO samples was performed using DESeq2. Gene set enrichment analysis (GSEA) was run using all genes against the Hallmark and KEGG gene sets using default parameters with the GSEApy package in Python (Fang et al., 2023).

### 3.10 Statistical analyses

Bayesian estimation to quantify the number of TSP niches was performed using R (The R Foundation for Statistical Computing). Statistical tests were done using Prism (V9.2.0; GraphPad Software), and significance was determined using an unpaired Student *t* test, one‐way analysis of variance (ANOVA) with Kruskal–Wallis multiple comparisons test or two‐way ANOVA with Sidak's multiple comparisons test, as indicated in the figure legends.

## AUTHOR CONTRIBUTIONS

JS, AV, ERR, and LIRE designed the experiments and wrote the manuscript. JS, AV, CS, HJS, and EP performed experiments and analyzed the data. BL, SAS, and CS performed statistical analyses. AK, ERR, and LIRE edited the manuscript.

## CONFLICT OF INTEREST STATEMENT

The authors declare no conflicts of interest.

## Supporting information


Data S1:

## Data Availability

For original data, please contact lehrlich@austin.utexas.edu or erichie@mdanderson.org. RNA‐seq data from this study are available at Gene Expression Omnibus (GEO) under accession number GSE230723.
