# Supplementary material for: The initial age‐associated decline in early T‐cell progenitors reflects fewer pre‐thymic progenitors and altered signals in the bone marrow and thymus microenvironments
Source: Aging Cell. 2023 May 23;22(8):e13870. doi: 10.1111/acel.13870 (PMC10410006; doi:10.1111/acel.13870)
Supplement: Supplementary file 1 — Data S1: [file ACEL-22-e13870-s001.pdf]

Supplementary Figures

Supplementary Figure 1

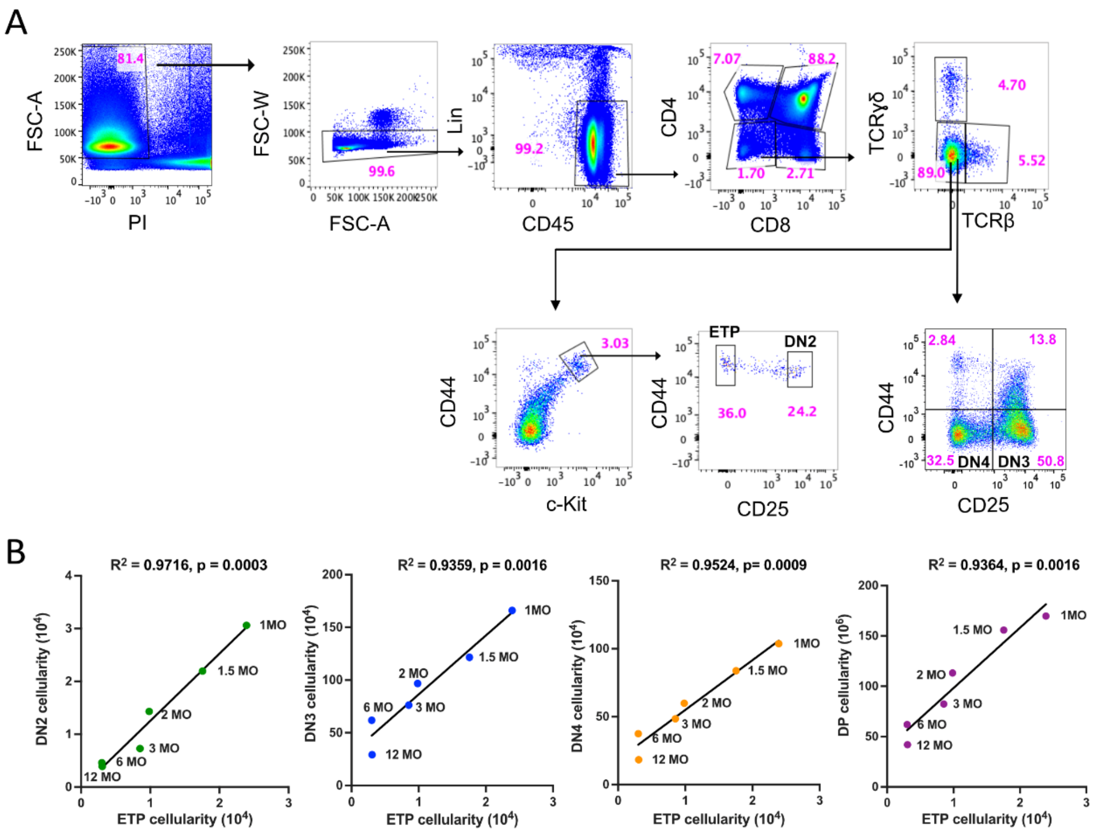

Supplementary Figure 1, related to Figure 1. Gating strategy for identification of thymocyte subsets and correlation of ETP cellularity with downstream thymocyte subsets. (A) Representative flow cytometry plots showing gating strategy for ETPs (Lin^- $c\text{-}kit^+$ $CD44^+$ $CD25^-$), DN2s (Lin^- $c\text{-}kit^+$ $CD44^+$ $CD25^+$), DN3s (Lin^- $c\text{-}kit^-$ $CD25^+$), DN4s (Lin^- $c\text{-}kit^-$ $CD25^-$), as well as DN, $CD4^+CD8^+$ DP, CD4SP, and CD8SP thymocyte subsets. DN subsets were identified within Lin^- $CD4^-$ $CD8^-$ $TCR\alpha\beta^-$ $TCR\gamma\delta^-$ thymocyte fraction. (B) Linear regression analysis of ETP cellularity and (from left to right) DN2, DN3, DN4 and DP cellularity at each age. R^2 = coefficient of correlation. Symbols represent data from an average of 7-14 mice at each age.

Supplementary Figure 2

A

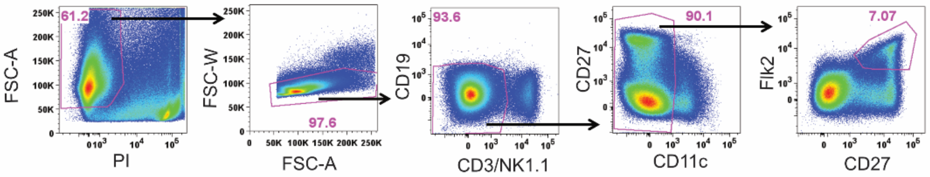

B

Parental, recipient and eight F1 donor strains for the multicongenic barcoding experiment

Parental Strains	CD45.1; Thy1.2	CD45.2; Thy1.2	GFP+; CD45.2; Thy1.2	CD45.2; Thy1.1
CD45.1; Thy1.2	(1) CD45.1; Thy1.2	(2) CD45.1; CD45.2; Thy1.2	(3) GFP+; CD45.1; CD45.2; Thy1.2	(4) CD45.1; CD45.2; Thy1.1; Thy1.2
CD45.2; Thy1.2		CD45.2; Thy1.2 (Recipient strain)	(5) GFP+; CD45.2; Thy1.2	(6) CD45.2; Thy1.1; Thy1.2
GFP+; CD45.2; Thy1.2				(7) GFP+; CD45.2; Thy1.1; Thy1.2
CD45.2; Thy1.1				(8) CD45.2; Thy1.1

C

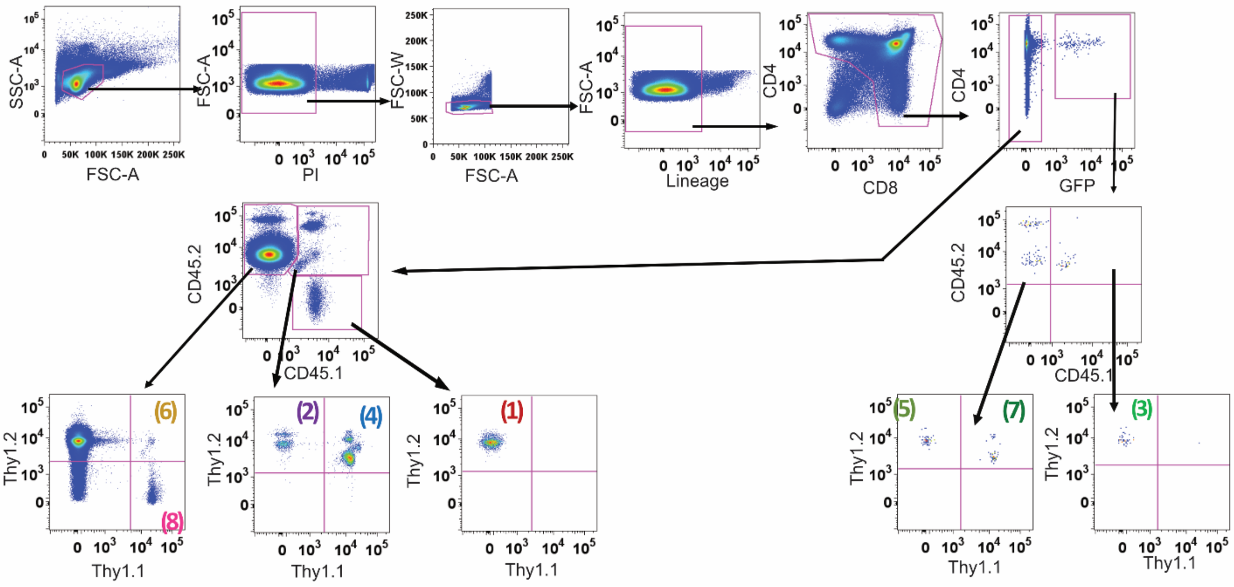

Supplementary Figure 2, related to Figure 2. Identification of donor strain-derived thymocytes in recipient thymuses following transfer of multicongenic progenitors. (A) Representative gating strategy used for sorting multicongenic Flk2^{+} CD27^{+} progenitors. BM from

all donors were depleted of mature lineages with antibodies against CD11b, CD11c, CD19, B220, Gr-1, NK1.1 and Ter-119. $CD3^+$, $NK1.1^+$ and $CD11c^+$ cells were excluded by gating and $Flk2^+$ $CD27^+$ progenitors were FACS sorted. (B) Eight distinct congenic F1 donor strains were generated by intercrossing CD45.1, CD45.2, GFP, and Thy1.1 strains. (C) Representative gating strategy to identify individual donor strains color-coded as in (B) 21d after i.v. transfer into non-irradiated 1MO, 3MO, 6MO and 12MO C57BL6/J recipient mice. The experiment was performed 4 times, with n = 10-12 recipients per age.

Supplementary Figure 3

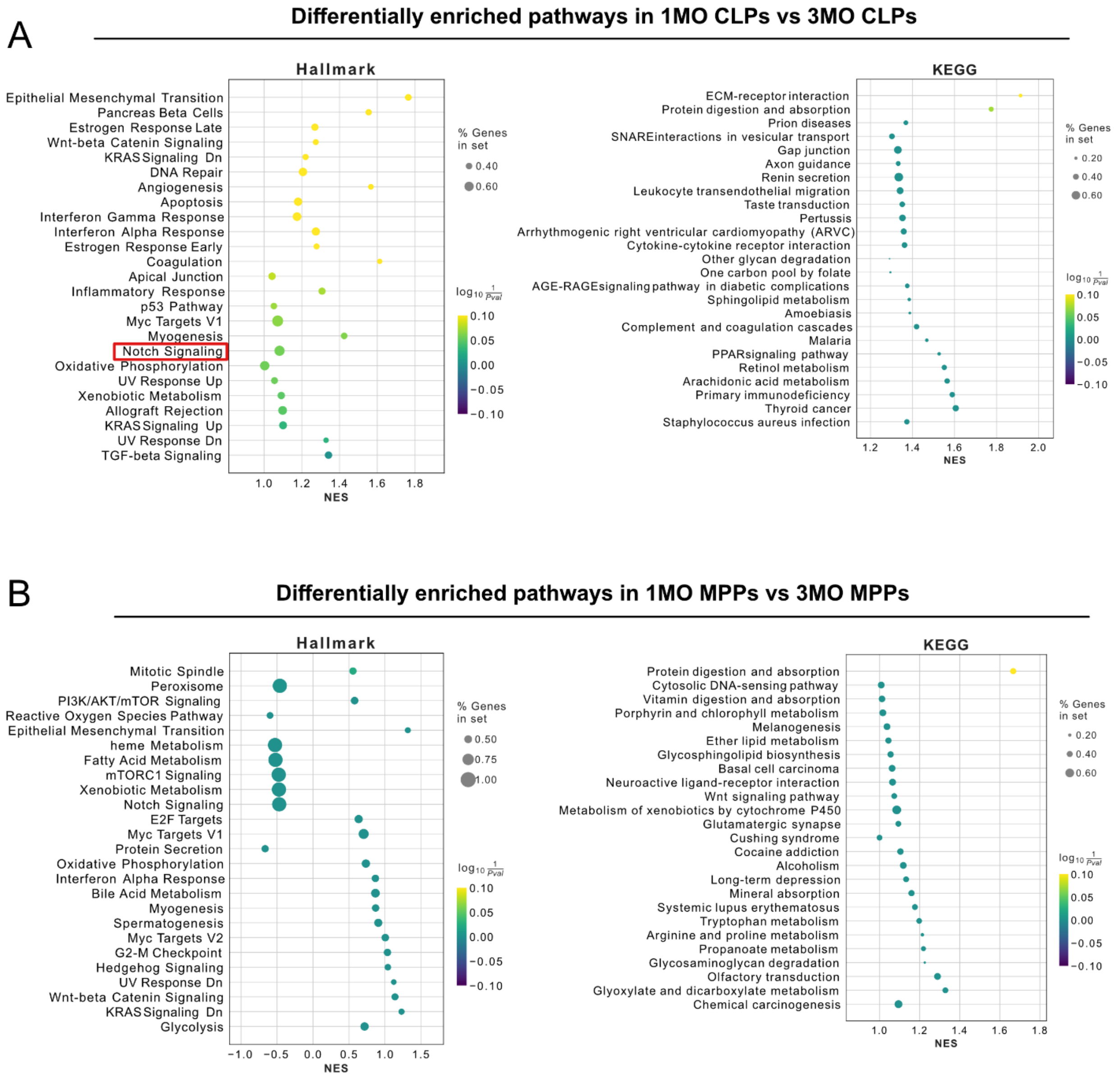

Supplementary Figure 3, related to Figure 5. Differentially enriched pathways in 1MO versus 3MO Ly6d$^-$ CLPs and Flk2$^+$ MPPs. (A-B) Dot plots representing the top 25 differentially enriched Hallmark and KEGG pathways, ranked by normalized enrichment scores (NES), upregulated or downregulated in (A) 1MO CLPs and (B) 1MO MPPs, compared to their 3MO counterparts, as identified by gene set enrichment analysis. Positive NES indicates pathways that are

upregulated in 1MO CLPs and MPPs, while a negative NES represents upregulation of the respective pathways in 3MO CLPs and MPPs. Data are averaged from 3 independent biological replicates of 1MO and 3MO CLPs and MPPs.

Supplementary Figure 4

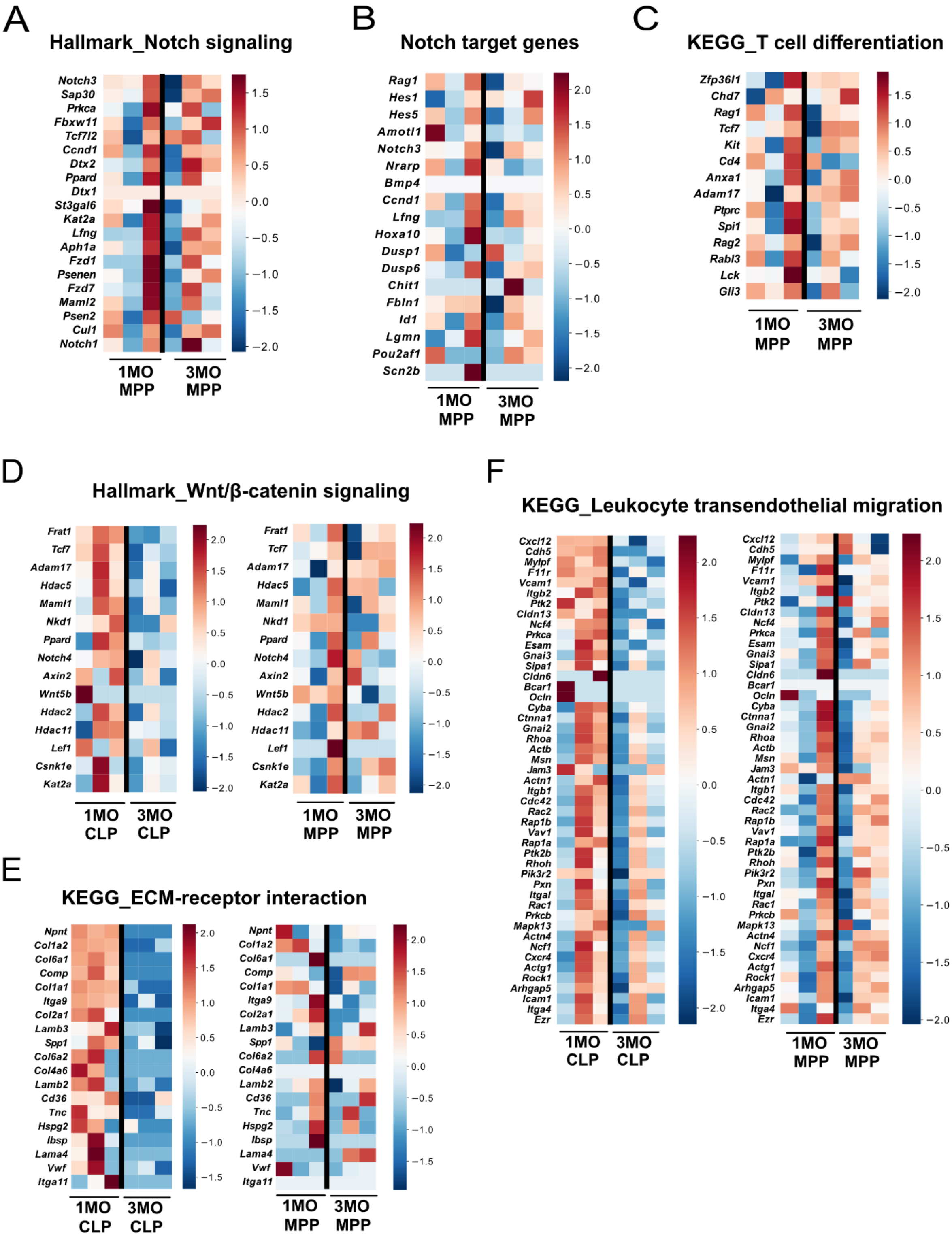

Supplementary Figure 4, related to Figure 5. Age-associated decline in enrichment of signaling and adhesion pathways in 3MO versus 1MO Ly6d^{-} CLPs. (A-C) Heatmaps show row-normalized z-scores of gene expression values indicating lack of enrichment of (A) the Notch signaling pathway genes (Hallmark), (B) Notch target genes, and (C) the T cell differentiation (KEGG) pathway in 1MO versus 3MO Flk2^{+} MPPs (N=3 independent experiments). (D-F) Heatmaps show row-normalized z-scores of gene expression values for genes associated with (D) Wnt/β-catenin signaling (Hallmark), (E) ECM-receptor interaction (KEGG), and (F) Leukocyte transendothelial migration (KEGG) pathways in 1MO and 3MO Ly6d^{-} CLPs (left) and Flk2^{+} MPPs (right). (A-E) Data are derived from 3 independent experiments.

Supplementary Figure 5

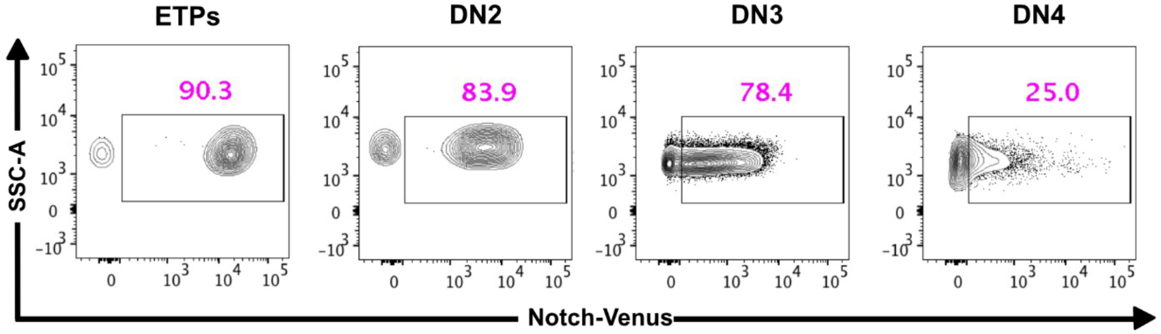

Supplementary Figure 5, related to Figure 6. Notch-Venus signaling reporter demonstrates faithful expression by immature thymocyte subsets. Representative flow cytometry plots show the frequency of cells undergoing Notch signaling as well as Venus expression levels in the Notch-Venus reporter mouse strain for the indicated thymocyte DN subsets.

Supplementary Table 1: List of Antibodies

Target	Fluorophore	Manufacturer	Clone	Dilution
B220	PE/Cy5	eBioscience	RA3-6B2	1:50
anti-BrdU	APC	BD Biosciences		1:100
Cleaved Caspase-3	BV421	Cell Signaling Technologies	D3E9	1:100
CD3	PE/Cy5	BioLegend	145-2C11	1:50
CD3	PE/Cy7	BioLegend	145-2C11	1:200
CD4	BV510	BioLegend	RM4-5	1:100
CD4	PE	BD Pharmingen	RM4-5	1:200
CD8	PE/Cy7	eBioscience	53-6.7	1:80
CD8	APC/Cy7	BioLegend	53-6.7	1:200
CD11b	PE/Cy5	Tonbo Biosciences	M1/70	1:50
CD11c	PE/Cy5	BioLegend	N418	1:50
CD11c	PerCP/Cy5.5	Tonbo Biosciences	N418	1:200
CD25	FITC	BD Pharmingen	7D4	1:40
CD25	AF700	BioLegend	PC61	1:800
CD25	Pacific Blue	BioLegend	PC61	1:50
CD27	APC	BioLegend	LG.3A10	1:200
CD27	PE/Cy7	BioLegend	LG.3A10	1:200
CD44	APC	BioLegend	IM7	1:50
CD44	FITC	Tonbo Biosciences	IM7	1:400
CD45	PerCP/Cy5.5	Invitrogen	30-F11	1:100
CD45	BV510	BioLegend	30-F11	1:100
CD45.1	APC	BioLegend	A20	1:200
CD45.2	PE/Cy7	BioLegend	104	1:150
c-kit (CD117)	APC/Cy7	BioLegend	2B8	1:40
EpCAM	APC	BioLegend	G8.8	1:300
Flk-2 (CD135)	PE	BioLegend	A2F10	1:200
Flk2 (CD135)	APC	BioLegend	A2F10	1:50
Gr-1	PE/Cy5	BioLegend	RB6-8C5	1:50
IL-7R (CD127)	Biotin	BioLegend	A7R34	1:100
Ly51	PE	BioLegend	6C3	1:300
Ly6D	PE	BioLegend	49-H4	1:200
I-A/I-E (MHC-II)	APC/Cy7	BioLegend	M5/114.15.2	1:200
NK1.1	PE/Cy5	BioLegend	PK136	1:50
NK1.1	PE/Cy7	BioLegend	PK136	1:200
Notch1	PE	BioLegend	HMN1-12	1:100
Sca-1	Pacific Blue	BioLegend	D7	1:200
TCRβ	AF700	BioLegend	H57-597	1:50
TCRβ	PE	BioLegend	H57-597	1:40
TCRγ/δ	PE	eBioscience	eBioGL3	1:40
Ter-119	PE/Cy5	eBioscience	TER-119	1:50
Thy1.1	AF700	BioLegend	OX-7	1:200
Thy1.2	BV510	BioLegend	30-H12	1:100
UEA-1	FITC	Vector Laboratories		1:500
Fixable viability dye-zombie red		BioLegend		1:1000
	Qdot®605	Invitrogen	Streptavidin	1:200
CD31	Pacific blue	Biolegend	390	1:200
